# Field validation and application of the luminex triplex HIV assay to estimate HIV prevalence and HIV-1 incidence in Nigeria

**DOI:** 10.1371/journal.pgph.0003455

**Published:** 2025-04-09

**Authors:** Ernest L. Yufenyuy, Olusola A. Akanbi, Vedapuri Shanmugam, Kelsie Decker-Pulice, Jeni Vuong, Mervi Detorio, Amy Zheng, Orji Bassey, Ado G. Abubakar, Oluwaseun Akinmulero, Mudiaga Esiekpe, Andrew Thomas, Iliyasu Abubakar Bichi, Israel Tamunonengiyeofori, Chinwe Ugwu, Evbuomwan Erasogie, William Nwachukwu, Nwando Mba, Ndidi Agala, Megan Bronson, Hetal K. Patel, Nnaemeka C. Iriemenem, Stacie Greby, McPaul I. Okoye, Mahesh Swaminathan, Bharat S. Parekh, Chikwe Ihekweazu

**Affiliations:** 1 Division of Global HIV & TB, Centers for Disease Control and Prevention, Atlanta, Georgia, United States of America; 2 Public Health Institute/Centers for Disease Control, Global Health Fellowship Program, Atlanta, Georgia, United States of America; 3 Nigeria Centre for Disease Control and Prevention, Abuja, Nigeria; 4 Division of Global HIV & TB, United States Centers for Disease Control and Prevention, Abuja, Nigeria; 5 Laboratory Quality Improvement Unit, Institute of Human Virology, Abuja, Nigeria; Universidad Autonoma de Baja California, MEXICO

## Abstract

HIV cross-sectional surveys require multi-layered testing with several tests to estimate HIV prevalence and HIV-1 incidence. We evaluated the performance and accuracy of the newly developed HIV Triplex assay to diagnose HIV-1 and HIV-2 and detect HIV-1 recent infections using plasma samples from the 2018 Nigeria AIDS Indicator and Impact Survey (NAIIS). Plasma samples from consenting HIV-positive (n=2,773) and a subset of HIV-negative samples (n=7,196), as determined by the national rapid testing algorithm, followed by Bio-Rad Geenius HIV-1/2 Supplemental Assay and Western Blot, aged 18 months - 64 years, were tested using the Luminex-based HIV Triplex assay. The assay classified specimens as HIV-1 positive, HIV-2 positive, dual (HIV-1 & 2) infections, or HIV-seronegative. All HIV-1 and dual infections were further classified as either HIV-1 recent (<6 months) or long-term (LT) based on mean fluorescent intensities and compared with the LAg-Avidity EIA as the reference. Multiplex results were analyzed and compared with the final NAIIS survey data for unweighted HIV prevalence and HIV-1 incidence. The diagnostic sensitivity and specificity of the HIV Triplex assay was 99.71% and 99.37%, respectively, with a *kappa* of 0.987 when compared to NAIIS survey results. Percent agreement between the HIV Triplex assay and the LAg-Avidity EIA for recent and LT classification was 98.86% with a kappa of 0.80 [CI: 0.71-0.89] and a Spearman-ranked correlation (ρ) of 0.689. A small number (n=45; 0.63%) of the subset of negatives tested were classified by the multiplex assay as either HIV-1 positive (n=35) or HIV-2 positive (n=10). Nevertheless, the HIV Triplex assay agreed with NAIIS HIV-negative survey results (99.37%). Using these results as they were, unweighted estimates of HIV prevalence for both HIV Triplex assay and NAIIS test results were similar (1.62% [95% CI: 1.56-1.68] and 1.60% [95% CI: 1.54-1.66], respectively) with overlapping confidence. After adjusting for viral load and anti-retroviral therapy, HIV-1 unweighted incidence for ages ≥15 years, using HIV Triplex assay data, was 0.70 per 1,000 [95% CI: 0.40-0.90]. This is similar to the unweighted incidence using the LAg-based RITA (recent infection testing algorithm) of 0.80 per 1,000 [95% CI: 0.60-1.10]. The HIV Triplex assay combines several assays in one, providing highly accurate results for estimating HIV prevalence and HIV-1 incidence in surveys. This assay has the potential to simplify cross-sectional surveys making them less expensive, easier, and quicker.

## Introduction

Human immunodeficiency virus (HIV), the causative agent of AIDS, has significantly contributed to global deaths for around 40 years [1–3]. Although AIDS-related mortality has declined since 2010, current estimates project that about 40 million people have died from AIDS-related illnesses [1], and about 39 million are living with HIV globally [1]. The decline in mortality is directly linked to highly active antiretroviral therapy (ART) that controls HIV viral load (VL) and disease but falls short of an HIV cure [4]. Despite current advances, up to 14% of those living with HIV do not know their status, and as many as 1.3 million new infections occurred in 2022 [1]. New infections, also called recent infections (<6 months), are the drivers of the HIV epidemic, and understanding the epidemiology of recent infections through surveys can facilitate global epidemic control.

National household-based serosurveys, such as the 2018 Nigeria HIV/AIDS Indicator and Impact Survey (NAIIS), are designed to estimate HIV prevalence and HIV-1 incidence in the general population for assessing the impact of HIV programs [5–8]. However, multiple laboratory methods are required in an algorithm and in sequential steps to achieve these major objectives. Using an algorithm in HIV diagnosis or recent infection detection increases the accuracy and positive predictive value of the results in both cases. However, using several assays to diagnose and confirm HIV serology, perform HIV serotyping, and distinguish recent infection from long-term infection is complex, costly, takes long hours, and laborious. This also inherently leads to additional sample volume requirements for the multiple testing steps. To improve the ease and accuracy of conducting surveys and estimating HIV incidence and prevalence, we developed a multiplex bead-based assay that combines multiple steps in HIV diagnosis and serotyping (in an algorithm-driven manner); and recency classification, all in a single assay (i.e., HIV Triplex assay).

Previously, the HIV Triplex assay was validated in the laboratory using a well-characterized worldwide panel of serum or plasma specimens [9]. This validation showed high diagnostic sensitivity and specificity and high agreement with the limiting antigen avidity enzyme immunosorbent assay (LAg-Avidity EIA assay) for recent infection classification, suggesting that the multiplex assay could be used as a single assay in surveys to estimate both HIV prevalence and HIV-1 incidence. However, its use in the field using cross-sectional specimens has not been validated. The purpose of this study was to evaluate the performance and accuracy of the HIV Triplex assay to detect HIV-1 and HIV-2 and distinguish recent and long-term HIV-1 infections and compare the multiplex-derived HIV prevalence and incidence estimates with those from NAIIS.

## Materials and methods

### Cross-sectional Specimens

NAIIS, a cross-sectional, household-based survey, used a 2-stage cluster design to obtain a nationally representative samples of individuals living in households. Cross-sectional specimens were obtained from the NAIIS and these specimens were accessed for this research activity on 16/06/2019. NAIIS methods have been described elsewhere [10]. The unlinked (no personal identifiable information) archived plasma specimens from consenting participants aged 18 months - 64 years, included all confirmed HIV-positives (n=2773), and a subset of HIV-negatives (n=7196) (by simple random selection of negative specimens from all states) to achieve a sample size of ~10,000 from a total of 170,946 specimens.

### Reference Standard

NAIIS HIV prevalence was estimated using Nigeria’s HIV serial rapid testing algorithm combined with Bio-Rad Geenius HIV-1/HIV-2 Supplemental Assay (Bio-Rad Laboratories, Hercules, CA, USA) and HIV-1 Western blot (Cambridge Biotech, Maryland, USA) as previously described [10]. The national testing strategy required a serial two-test algorithm with Determine HIV-1/2 rapid antibody test (Abbott Laboratories, Abbott Park, IL, USA), followed if reactive, by the UniGold Rapid HIV Test (Trinity Biotech PLC, Bray, Ireland). Specimens with two consecutive HIV reactive results on both tests were considered HIV-positive. Those with discordant results (Determine reactive and UniGold non-reactive) were tested using Stat-Pak (Chembio Diagnostic Systems, Medford, NY - USA) as the “tiebreaker.” The serial algorithm was conducted at the household. Specimens that were non-reactive on Determine or reactive on Determine but not on UniGold and Stat-Pak were considered HIV-negative. HIV-positives, as classified by national algorithm, were further confirmed, and serotyped using the Bio-Rad Geenius HIV-1/2 Supplemental Assay at the satellite laboratory. Specimens that were positive by the national algorithm but negative or indeterminate by Geenius were further tested by HIV-1 Western Blot (Cambridge Biotech, Maryland, USA) or HIV-DNA PCR for final HIV status determination.

**Fig 1:**
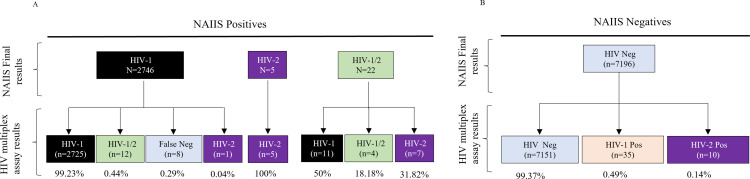
Shows NAIIS final diagnostic and serotyping results alongside HIV multiplex diagnostic and serotyping results. The figure shows NAIIS samples that were tested using the HIV Luminex multiplex assay. NAIIS positive results were determined using the national algorithm and confirmed with either Geenius, Western Blot or DNA PCR. On the other hand, NAIIS negative results were also determined using the national algorithm, Geenius confirmatory test, DNA PCR and Western Blot (used to resolve specimens that were Geenius indeterminate).

For incidence estimation, confirmed HIV-1 positives and HIV-1/2 dual Infections (confirmed using Geenius, Western Blot (WB), and/or HIV DNA PCR) were tested with the limiting antigen avidity enzyme immunosorbent assay (LAg-Avidity EIA) (Sedia Biosciences, Portland, OR, USA) to distinguish recent from long-term HIV-1 infection. VL was conducted on all HIV-1 positive specimens, and RITA (recent infection testing algorithm) recents were LAg-recent cases with VL ≥ 1000 copies/mL in the absence of antiretroviral (ARV) metabolites. This number was used to estimate HIV incidence using a mean duration of recent infection of 130 days. Note that all HIV positives in NAIIS were tested for ARVs. A summary of NAIIS diagnostic reference results for this validation is shown in Fig 1 (top panel).

### Multiplex assay

The HIV Triplex assay was performed as described earlier [9] but with slight modifications. Two antigens, recombinant p24-gp41 (Bioprocess, Inc., Australia), and HIV-2 immunodominant peptide, were coupled to two different microspheres (bead region (BR)-12 and BR-14, respectively) at saturating concentrations, while the recombinant gp41 rIDR-M antigen was coupled to a third microsphere (BR-13) at limiting concentrations, as described [9]. The coupled beads were mixed and resuspended in a buffer, and ~3,000 beads of mixture were dispensed on each well in a 96-well plate, dried, sealed and stored at 4°C until needed. The plates with dried beads were verified to have the same mean fluorescence intensity (MFI) as freshly prepared beads using quality control specimens (manuscript in preparation). Before use, the dried-coupled-beads were rehydrated in 100 µl of wash buffer while shaking for 10 min at room temperature, followed by 2 wash cycles before incubating with diluted samples and controls (HIV-negative, HIV-1 recent, HIV-1/2 long-term) while shaking at room temperature. The controls were run in duplicate (negative control) or in triplicate (all other controls) on each plate. All specimens were initially tested in singlets and confirmatory testing in triplicate. Confirmatory testing was performed for specimens screening with high MFI (>1,500 on BR-12 with p24-gp41 antigen for diagnosis or >1,000 on BR-14 on BR-14 with HIV-2 IDR peptide for serotyping), while repeat testing was performed in triplicate on discordant samples between NAIIS final and the HIV Triplex assay results.

### Plate validation, interpretation of the results, and data analysis

Quality control specimens were included in each plate whose passing criteria were based on the MFI of each bead region (Table 1). Test runs were considered valid only if the following conditions were met: 1) if the negative control had MFI less than 1,000 on all bead regions, 2) if HIV-1 control had an MFI between 25,000 and 40,000 in bead region 12 (p24-gp41 antigen), MFI between 750 and 1,500 in bead region 13 (rIDR-M), and an MFI less than 500 in bead region 14 (HIV-2 IDR), and 3) if HIV-1/2 control had MFI between 25,000 and 40,000 in bead region 12(p24-gp41 antigen), MFI between 1,500 and 3,500 in bead region 13 (rIDR-M), and an MFI between 15,000 and 30,000 in bead region 14 (HIV-2 IDR). This is summarized in Table 1. HIV multiplex data interpretation was based on the MFI using previously determined cutoffs shown in Table 2 under cutoff MFI. The cutoff for an HIV-1 positive diagnosis using the p24-gp41 fusion protein is MFI ≥ 4,000, HIV-2 positive diagnosis using HIV-2 IDR is MFI ≥ 2,000, and recent infection classification is MFI ≤ 1,250 for the rIDR-M. However, it should be noted that diagnosis, serotyping, and recency classification in this multiplex format relies on an algorithm that utilizes the different antigens as illustrated in Table 2. The final interpretation of HIV-negative, HIV-1 positive only, HIV-2 positive only, HIV-1/2 dual infection, and recent or long-term infections relied on an algorithm that utilized a combination of antigens with median MFIs resulting from the bead regions as previously reported [9] and as shown in Table 2. Prevalence of HIV-1, HIV-2 and dual infections were calculated using number positive in each category divided by total specimens in the NAIIS survey. HIV-1 incidence was calculated using numbers classified as RITA-recent cases after adjusting for viral load (≥1,000 copies/mL) and absence of ARV and an MDRI of 130 days and FRR of 0.0. All results were compared with the diagnostic and incidence testing results from NAIIS using GraphPad descriptive statistics (https://www.graphpad.com/quickcalcs/kappa1/, accessed September 15, 2023) to calculate sensitivity, specificity, confidence intervals, percent agreements, kappa values, and Spearman ranked correlations, as appropriate. The sensitivity and specificity of the multiplex assay were calculated using true positives, false positives, true negatives and false negatives as determined by the NAIIS survey. A receiver operating characteristic (ROC) curve was also generated to evaluate the diagnostic performance of the assay over a range of possible cutoff points, and an optimal cutoff point was determined. Additionally, the area under the ROC curve was calculated. Agreement between recent and long-term were assessed using a two-by-two table and *Kappa* value to compare the multiplex assay recency classification with recency classification using the LAg-Avidity EIA. Results were also used to calculate assay-derived unweighted HIV prevalence and incidence.

**Table 1:** Raw mean fluorescent intensity ranges for quality control samples used on each plate. The fluorescent intensities for each of the controls must fall within the minimum and maximum ranges for each bead region for the plate to be valid.

Specimen Type	Bead region	Antigen	Purpose	Min. MFI	Max. MFI
**Negative QC**	BR-12	p24-gp41	**HIV Diagnosis**	0	1,000
	BR-13	rIDR-M	**Recency**	0	1,000
	BR-14	HIV-2 IDR	**HIV Serotyping**	0	1,000
**HIV-1 QC**	BR-12	p24-gp41	**HIV Diagnosis**	25,000	40,000
	BR-13	rIDR-M	**Recency**	750	1,500
	BR-14	HIV-2 IDR	**HIV Serotyping**	0	500
**HIV-1/2 QC**	BR-12	p24-gp41	**HIV Diagnosis**	25,000	40,000
	BR-13	rIDR-M	**Recency**	1,500	3,500
	BR-14	HIV-2 IDR	**HIV Serotyping**	15,000	30,000

**Table 2:** Raw mean fluorescent intensity ranges for quality control samples used on each plate. The table shows antigens used, the MFI cutoff for the antigen and how the antigens are used in combination to define the HIV status of each specimen. For examples, HIV-½ positive infection classification uses p24-gp41, rIDR-M antigens and the HIV-2 IDR peptide in an algorithm driven by the MFI from each bead.

Specimen Type	Bead region	Antigen	Cutoff MFI
**HIV-1 Diagnosis**	BR-12	p24-gp41	4,000
**HIV-2 Diagnosis**	BR-14	HIV-2 IDR	2,000
**HIV-1 Recency Classification**	BR-13	rIDR-M	1,250

### Ethical approval

The amended NAIIS protocol was approved by the Institutional Review Board of the University of Maryland, Baltimore and the United States Centers for Disease Control and Prevention in Atlanta, GA USA, with protocol number 7103, and the Nigerian National Health Research Ethics Committee.

## Results

### Diagnostic performance of the HIV triplex assay

A total of 9,969 specimens were tested on the HIV Triplex assay. A total of 2,765 of the 2,773 NAIIS HIV-positives also tested HIV-positive by the HIV Triplex assay giving a sensitivity of 99.71% [95% CI: 99.43-99.88]. On the other hand, 7,151 out of 7,196 specimens were confirmed as HIV-negative by the multiplex test, giving a specificity of 99.37% [95% CI: 99.16-99.54]. The results are summarized in Fig 1,Tables 3 and 4. Based on these samples and population tested, other test parameters such as the positive predictive value (PPV), negative predictive value (NPV) and percent accuracy of the HIV Triplex assay were calculated at 98.4% (95% CI: 97.87%-98.80%), 99.89% (95% CI: 99.78%-99.94%), and 99.47% (95% CI: 99.31%-99.60%) respectively.

**Table 3:** Comparing the performance of the diagnostic and serotyping components of the multiplex assay with results from NAIIS: Determine, Unigold, StatPak, Geenius, Western Blot and HIV DNA PCR.

	NAIIS Final Results-All Results			
Multiplex Assay		Pos	Neg	Total
	Pos	**2,765**	45	2,810
	Neg	8	**7,151**	7,159
	Total	2,773	7,196	**9,969**

**Fig 2:**
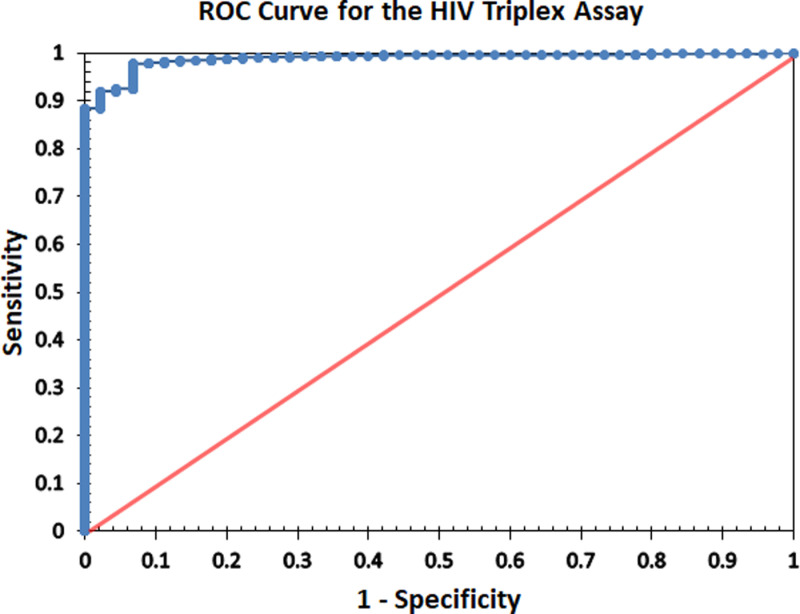
Receiver operating characteristics (ROC) curve analysis for the mean fluorescence intensity (MFI) of the HIV Triplex assay.

NAIIS classified five specimens as HIV-2, 22 as HIV-1/2, and 2,746 HIV-1 positive (Fig 1A, top panel). The Triplex assay classified the five HIV-2 specimens from NAIIS as HIV-2. However, of the 22 specimens classified as dual infections by Geenius, the Triplex assay classified only four of these specimens as dual infections, 11 as HIV-1, and seven as HIV-2 infections (Fig 1A, bottom panel). Of the 2,746 HIV-1 specimens, the HIV Triplex assay classified 2,725 specimens (99.2%) as HIV-1, 12 (0.4%) as dual infections, 8 (0.3%) as HIV-negative, and one as HIV-2. The HIV Triplex assay results were also used to calculate assay-unweighted HIV prevalence in Nigeria at 1.62% (Table 5). These calculations were based on a predetermined assay cutoff of 4000 MFI. However, we performed receiver operating characteristics (ROC) curve analysis on a range of possible MFI cutoff points. The primary objective of this additional analysis was to establish if the predetermined cutoff point was the most optimal cutoff value for determining HIV infection. A ROC curve was plotted as shown in Fig 2. The area under the curve (AUC) was calculated at 0.988 (95% CI:).981-0.995) indicating excellent diagnostic performance of the test. A cutoff of 4000MFI, which has been previously validated has a sensitivity of 99.6% and is an appropriate cutoff based on the ROC curve. It should be noted that cutoffs of 3000 MFI to 5000 MFI had similar sensitivities ranging from 99.6% to 99.5%. However, the 1-sensitivity values decreased significantly with cutoffs greater 4000 MFI.

### Recency performance of the HIV Triplex assay to distinguish recent from LT infections

**Fig 3:**
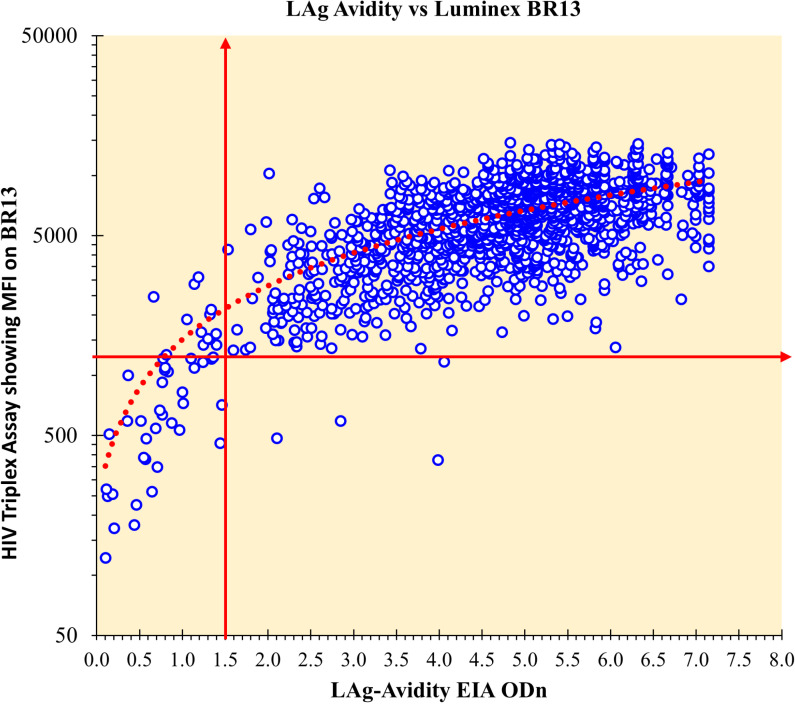
A scatter plot showing the correlation between the LAg-Avidity EIA and the HIV Luminex multiplex assay with the line of best fit (dotted red line); and the separation between recent and LT infections shown for each test using the vertical and horizontal arrows: for LAg-Avidity EIA at 1.5 ODn and at 1250 MFI.

During NAIIS, the LAg-Avidity EIA was performed on confirmed HIV-1 and HIV-1/2 specimens for participants 15-64 years old. There were 41 participants that were HIV positive but <15 and they were excluded from recent infection classification. Of the 2,727 confirmed HIV-1 or HIV-1/2 specimens ≥15 years old, the LAg-based RITA (LAg-recent+ VL≥1,000 + no ART detected) classified 48 specimens as recent infections. However, 2,746 specimens were classified by the HIV Triplex assay as HIV-1 or HIV-1/2, of which 39 were further classified as recent for 15-64 years old by the HIV Triplex-RITA (Triplex-recent +VL≥1,000, and no ART detected) (Table 4). The calculated overall percent agreement between the LAg-RITA and Triplex-RITA is 99.37%, a kappa of 0.801, and a Spearman ranked correlation of 0.689 (Table 4, Fig 3). Moreover, the unweighted LAg-RITA derived incidence calculated from NAIIS was similar to that of the Triplex-RITA at 0.08% [95% CI: 0.06%-0.11%] and 0.07% [95% CI: 0.04%-0.09%], respectively (Table 5).

**Table 4:** Comparison of the LAg Avidity EIA at 1.5ODn and the Multiplex assay at 1250 MFI for VL≥1000. Concordant results between the two assays are shown in red. Note that participants <15 years old were removed from recency classification.

	LAg-EIA at 1.5 ODn, VL ≥ 1,000			
Multiplex Assay		Recent	LT	Total
	Recent	**35**	4	39
	LT	13	**2,659**	2,672
	Total	48	2,663	**2,711**

**Table 5:** Comparison of unweighted HIV prevalence and HIV-1 incidence estimates from the NAIIS survey with Multiplex assay-based testing. Prevalence reference was based on final HIV status by Geenius, HIV-1 Western blot and DNA PCR while HIV-1 incidence was estimated using the LAg-Avidity EIA + VL+ART algorithm or Triplex Assay + VL +ART algorithm.

	Prevalence [95% CI]	Incidence/100 PY [95% CI]
NAIIS	1.60 [95% CI: 1.54-1.66]	0.080 [95% CI: 0.06-0.11]
Multiplex Validation	1.62 [95% CI: 1.56-1.68]	0.070 [95% CI: 0.04-0.09]

### Serotyping performance of the HIV Triplex assay to differentiate HIV-1, HIV-2, and HIV-1/2 infections

Based on the Triplex assay algorithm, there were 23 HIV-2 compared to only five HIV-2 from the NAIIS final results. Additionally, NAIIS identified 22 HIV-1/2 dual infections, while HIV Triplex assay identified 16 HIV-1/2 (Fig 1A). All specimens classified by NAIIS as HIV-2 were also classified by the Triplex assay as HIV-2. However, an additional 18 HIV-2-only specimens were also identified; seven from the NAIIS HIV-1/2 pool, one that was Geenius indeterminate/WB positive from the HIV-1 pool, and 10 from the NAIIS HIV-negative pool (Fig 1). We further analyzed the results of the eight HIV-2 identified by Triplex assay from the HIV-1/2 pool, comparing their reactivity in the LAg-Avidity EIA (Table 6). The results showed very low normalized optical densities suggesting the absence of HIV-1 infections because the antigen used in LAg is not expected to bind to HIV-2 antibodies. Only 4 (18%) of the 22 specimens identified in the NAIIS final results as dual infections were determined to be HIV-1/2 by the Triplex assay algorithm, while the remainder were either HIV-2 (n=7) or HIV-1 (n=11). The HIV Triplex assay algorithm reclassified 12 specimens as HIV-1/2 dual infections NAIIS classified as HIV-1 infections. The agreement between the HIV multiplex assay and Geenius in classifying HIV-2 and HIV-1/2 was low at 23%. The proportion of HIV-2 in the NAIIS and HIV Triplex results are 0.18% and 0.82%, respectively, representing a more than 4-fold increase and not extrapolated to all NAIIS negative samples.

**Table 6:** Comparing HIV Triplex Assay serotyping results with those of Geenius and the LAg-Avidity EIA showing very low normalized optical density (ODn) and target not detected (TND) on HIV-1 viral load for a subset of specimens classified by the HIV Triplex assay as HIV-2. The table shows high MFI for HIV Triplex Assay, low ODn on LAg-Avidity EIA suggestive of HIV-2 infections.

Serotyping MFI Triplex assay	Triplex assay Interpretation	Geenius Interpretation	LAg-Avidity EIA ODn	HIV-1 Viral Load
28,340	HIV-2	HIV-2	0.052	TND
11,563	HIV-2	HIV-1/2	0.064	TND
20,800.5	HIV-2	HIV-1/2	0.272	TND
17,158	HIV-2	HIV-1/2	0.099	TND
29,959.5	HIV-2	HIV-2	0.197	TND
21,270	HIV-2	HIV-1/2	0.064	TND
22,162	HIV-2	HIV-1/2	0.110	TND
13,740	HIV-2	HIV-2	0.428	TND
4,762	HIV-2	HIV-2	0.103	TND
15,483	HIV-2	HIV-2	0.156	TND
15,295	HIV-2	HIV-1/2	0.353	TND
2,529	HIV-2	HIV Indeterminate	0.235	TND
17,998	HIV-2	HIV-1/2	0.213	TND

## Discussion

Our results describe the first field application use of the HIV Triplex assay for the estimation of HIV prevalence and incidence using cross-sectional specimens. The HIV Triplex assay-based unweighted prevalence and incidence estimates compared similarly with NAIIS algorithm unweighted estimates (Tables 3 and 4). The similarities in the results are remarkable and indirectly validate many aspects of the survey and the assay.

The data reported here suggest that the HIV Triplex assay can provide estimates of HIV prevalence and incidence like those obtained from a serial testing algorithm using multiple assays. Although performed as a single assay, the Triplex assay results are still algorithm-driven within the assay and can simultaneously diagnose, serotype, and separate recent HIV-1 infections from long-term infections. The results are highly consistent with NAIIS final results classification with additional information inferred from the HIV Triplex assay. With a sensitivity of 99.71% and a specificity of 99.37% (Table 3), the HIV Triplex assay qualifies to adequately make clinical decisions and calculate survey estimates. ROC curve analysis results further show that the cutoff of 4000 MFI is the most optimal to separate positive from negative HIV infections. This extremely high cutoff value is optimized to maximize both sensitivity and specificity. These results indicate that the HIV Triplex assay can simplify surveys making the testing more efficient while saving sample volume and labor from multi-layered testing.

The sensitivity of the HIV Triplex assay was high at 99.71% and consistent with previous findings, while the specificity was slightly lower at 99.37% compared to previous findings [9]. However, the specificity results still meet WHO pre-qualification performance criteria of >98%. The high sensitivity and specificity may result from solution-based kinetics with continuous shaking during incubations and washes. Presumably, shaking during incubations increases sensitivity by allowing for mixing and multiple chances of all epitopes to encounter binding sites. In contrast, the shaking during the wash steps increases specificity by preventing non-specific binding from remaining bound to the beads. It is known that tests such as EIAs are a bit more sensitive in picking up early seroconverters compared to HIV rapid tests [11]. Similarly, it is possible that Triplex assay can also identify early seroconverters missed by rapid tests. The Triplex assay classified a total of 45 NAIIS HIV-negative specimens as HIV positive. Western Blot data was available for 13 of the 45 specimens, which had MFIs ranging from 4,971 to 19,179, and 5 as HIV-2 with MFIs ranging from 2,164 to 19,549. Seven out of eight HIV-1 were NAIIS WB indeterminate, with the most common bands being p24, p17, p51, p66 in different combinations without envelope. Also, all seven were NAIIS Geenius indeterminate, and one specimen was negative by both Geenius and WB. Two of the 5 HIV-2 were WB indeterminate, and two were also Geenius indeterminate by NAIIS. Interestingly, two of the WB indeterminate specimens were Geenius negative and vice versa. Only Determine results were available for the other 32 results that were positive by the HIV Triplex Assay but negative by NAIIS, with MFIs ranging from >4,000s to ~23,000. Thirteen out of 32 had MFI>7,000, while 9 had MFI that were between 5,000 and 7,000. This shows that most of these specimens did not have borderline MFI although additional testing could not be done to confirm their HIV status. Identification of additional HIV positives (n=45) by HIV Triplex assay warrants additional investigation. Although the Triplex assay demonstrates a high PPV of 98.4%, the results require careful interpretation, as diseases prevalence is a critical factor in determining their true significance. The PPV is highly sensitive to changes in disease prevalence-when the prevalence increases, the PPV also increases, and vice versa. In this study, the relatively small sample size inflated the diseases prevalence to approximately 28% which may have led to an overestimation of the assay’s accuracy. Therefore, caution is necessary when generalizing these findings to other settings with different prevalence rates.

The agreement between the HIV Triplex assay and the LAg-EIA in classifying recent and long-term infections was quite high (99.37%), with a ρ-value of 0.689. A few specimens changed classification from recent to LT or from LT to recent. Still, this observation was limited to specimens near the cutoff for both the Triplex assay and LAg-Avidity EIA. LAg-Avidity EIA and the Triplex assay accurately classified most specimens on ART as LT despite having a VL <1,000 copies/ml or target not detected (TND). In most cases, HIV-positives who are <15 years old are perinatally infected and assumed to be LT infections and are not tested by the LAg-Avidity EIA. However, the results of participants <15 were inferred from the HIV Triplex assay. All 41 specimens testing HIV-positive from NAIIS, from people aged 2-14 years, also tested HIV-positive on the HIV Triplex assay, and 39 were classified as LT. The Triplex assay classified two specimens as recent (both less than 15 years old with a VL = 15,013 copies/ml and 132, 796 copies/ml). No additional clinical data was available to understand the nature of these infections, suggesting that these individuals may be perinatally infected and failing drug treatment [12]. The results overall support the assumption that adolescents are an unlikely source of recent infections when perinatally infected and are unlikely to meaningfully contribute to areas of active infection and transmission however, these observations should be interpreted cautiously [13–16].

The HIV Triplex assay identified more HIV-2 specimens (n=23) and fewer dual infections compared to the Geenius HIV-1/2 Supplemental confirmatory assay. Specifically, only four of the 22 samples (18%) identified by Geenius as dual infections were classified by the HIV Triplex assay as dual, and seven were classified as HIV-2 by Triplex assay (Fig 1). The other 11 were classified as HIV-1 by the Triplex assay. We further showed that these 7 specimens, plus one more additional that was originally classified as HIV-1 by Geenius, could not bind to the rIDR-M, which is specifically designed to bind only HIV-1 (Fig 1 and Table 6). Interestingly, HIV-1 VL results (where available) showed target not detected (TND) for all 8 specimens suggesting that these could be true HIV-2 specimens although HIV-2 molecular testing was not performed. Additionally, the HIV Triplex assay classified 12 specimens as dual infections that were classified by the Geenius assay as HIV-1 only. These results suggest that while the diagnostic agreement between the two tests is very high (>99.7%), the serotyping agreement is not.

One limitation of the Geenius confirmatory assay is the cross-reactivity between HIV-1 and HIV-2 antibodies that limit differentiation between HIV-1 and HIV-2. Bio-Rad’s clinical evaluation of Geenius’s HIV-2 diagnostic and differentiation capacity showed that only 77/200 known HIV-2 samples were classified as HIV-2 only. At the same time, the remainder were interpreted as HIV-2 with HIV-1 cross-reactivity (108/200), HIV dual (untypable) (12/200), or HIV-2 indeterminate (3/200) [17]. Previous reports from other evaluations cited the Geenius assay having a lower differentiation capacity than Multispot and HIV-2 WB [18–19]. Our previous laboratory evaluation of the HIV Triplex assay correctly classified 30/31 well-characterized HIV-2 specimens [9]. The one misclassified specimen tested HIV-negative on the HIV Triplex assay. HIV typing, accorded by the HIV Triplex assay utilizes an algorithm driven by binding to the three antigens used: p24-gp41, HIV-2 IDR, and rIDR-M. The p24-gp41 binds predominantly HIV-1 but could have immunological cross-reactivity with HIV-2 due to p24. It is important to note that rIDR-M is derived from the immunodominant region of gp41 in group M HIV viruses and is designed to bind to HIV-1 antibodies exclusively. The HIV-2 IDR is a peptide derived from the immunodominant region of gp36 with high specificity for HIV-2. This combination of binding the antibodies to these antigens, or lack of binding thereof, provides highly precise differentiation and classification of HIV-1, HIV-1/2, and HIV-2 specimens. Additionally, specimens identified as HIV-2 by the HIV Triplex assay have all been mapped to geolocation clusters for HIV-2 infections in Nigeria as determined by previous molecular methods studies [20]. Put together; we think the HIV Triplex assay has a higher differentiation and serotyping capability compared to its Geenius HIV-1/2 counterpart. Future studies will seek to evaluate the Triplex assay’s serotyping ability in comparison with other HIV-2 tests.

The reference standard in this field validation is not from a single test but from a standard national HIV serial rapid testing algorithm coupled with confirmatory testing on Geenius HIV-1/2 supplementary assay and resolution testing using WB and HIV DNA PCR. This also suggests that the actual sensitivity and specificity of the HIV Triplex assay are higher than reported here. Our previous findings on the development of the HIV Triplex assay determined the sensitivity and specificity of the assay to be 99.7% and 99.7%, respectively. These results are consistent with an expected higher specificity for the current evaluation. Therefore, as a standalone assay, the HIV Triplex assay has high sensitivity and specificity, however we recognize that HIV diagnosis cannot be made using a single assay. Our future work on the HIV Triplex assay aims to significantly enhance its diagnostic capabilities by incorporating additional biomarkers, enabling an algorithm-driven approach for more accurate HIV diagnosis. This expansion will substantially improve the PPV in low prevalence populations, addressing the critical challenge in HIV testing. By integrating multiple biomarkers into the multiplex assay, we can generate a comprehensive set of results from a single test, effectively simulating the diagnostic power of multiple sequential tests. This approach will optimize both diagnostic accuracy and efficiency, making it a more robust tool for HIV detection.

One limitation of our study is that we did not test all the negative samples (n=170, 946) from NAIIS but only 7,196 (4.2%) of the negatives. Another limitation is that we could not use molecular testing to ascertain the status of the specimens identified as HIV-2. These samples have been thawed several times and are no longer ideal for molecular testing.

The development and use of the LAg-Avidity EIA and Asante HIV-1 Rapid Recency Assay which detect recent infections have propelled the field of HIV surveys and surveillance further. These assays have performed relatively well with other diagnostic assays and tools. However, because of the many steps involved, cost, labor, and time associated with these assays, the HIV Triplex assay may serve as an attractive alternative since it combines multiple assays in one, providing highly accurate results for the estimation of HIV-1 prevalence and incidence and HIV-2 prevalence, with high diagnostic and potentially serotyping accuracy. Although the Luminex Triplex HIV assay meets WHO pre-qualification standards for HIV diagnosis and serotyping, WHO currently lacks a pathway for prequalifying tests for recent infection surveillance or incidence estimation. As a result, not all components of the assay can meet WHO prequalification criteria. Nevertheless, this assay can simplify surveys making them less expensive, easier, and quicker.

## Supporting information

S1 Data Public available data on NAIIS multiplexing. (XLSX)
